# Knowledge and Practice of Parents Towards Measles, Mumps and Rubella Vaccination

**DOI:** 10.21315/mjms2022.29.3.9

**Published:** 2022-06-28

**Authors:** Siti Zuhaida Hussein, Nuraina Mardia, Mastura Amirah, Rosnita Hashim, Suraya Hanim Abu Bakar

**Affiliations:** Nursing Department, Faculty of Medicine, Universiti Kebangsaan Malaysia, Cheras, Kuala Lumpur, Malaysia

**Keywords:** vaccination, measles, mumps, rubella, MMR immunisation, children

## Abstract

**Background:**

Measles-mumps-rubella (MMR) vaccination is the safest and most effective way of protecting children from three potentially fatal diseases and yet, high numbers of children remained unimmunised.

**Objective:**

The study aimed to determine the parents’ knowledge and practice regarding MMR vaccination and examine its associations.

**Methods:**

This is a cross-sectional study conducted among the 202 parents in Selangor Malaysia using simple random sampling. The parents’ knowledge and practice regarding MMR vaccination were measured using the modified self-administered questionnaire developed by Awadh et al. The data were analysed using the Statistical Package for the Social Sciences Statistic for Windows (version 23.0) and the significant level (*P*) was set at *P* < 0.05.

**Results:**

Overall, 53.5% of parents had low knowledge of MMR vaccination, 35.1% had moderate knowledge and 11.4% had high knowledge of MMR vaccination. Meanwhile, 54.5% had good practice of MMR vaccination and 45.4% reported they had not practised immunising their children with the MMR vaccine. The number of respondents who had a higher level of knowledge and good MMR vaccination practices was 20.0%. There is a significant association between parental knowledge of MMR vaccination and the practice of immunising their children with the MMR vaccine (*P* > 0.001). Besides, there was a significant association between the level of knowledge on MMR vaccination with parental education background (*χ*^2^ = 12.06; *P* = 0.002), number of children (*P* = 0.040) and a working family (*P* = 0.030). The Pearson’s chi-squared test showed a significant association between gender (*P* = 0.040), the number of children (*P* = 0.05) and level of practice of MMR vaccination.

**Conclusion:**

The majority of parents have low knowledge of MMR vaccination but they practice good MMR vaccination. It is necessary to consider parents’ level of knowledge when planning the health promotions regarding MMR vaccinations.

## Introduction

Worldwide, the measles-mumps-rubella (MMR), a combination vaccination were highlighted to be the most effective disease prevention for controlling and eliminating life-threatening diseases in all stages of human life (1–3). Hence, the World Health Organization (WHO) demanded that all countries include the MMR vaccination as an element of the National Immunisation Programme. Two doses are given at the age of 12 months old and 7 years old.

Several media and experts from various fields recently discussed the MMR vaccines that could boost an individual’s immunity. It may be effective and have protective value against infection from SARS-CoV-2 and coronavirus for a limited period (4). In addition, some researchers stated these childhood MMR vaccines provided immune responses to viruses in the specific vaccines and responded to other viral infections in general (4). Thus, it might fight against similar viral infections likes COVID-19. However, the study on MMR vaccines and COVID-19 is still in the trial and the results have not been confirmed.

Unfortunately, because of the COVID-19 epidemic, the whole world is experiencing difficulties in achieving the target of the immunisation programme (1, 5). According to WHO (5), the decline in immunisation coverage is an exceedingly dangerous situation and it has happened for the first time since the early 90s. Indeed, some developed countries had lost their ‘measles free’ status, like the United Kingdom and Greece (6). Statistical data for 2018 shows the percentage of the first dose of MMR vaccine among children aged 2 years old in England has been the lowest since 8 years ago (7). Indeed, this percentage decreases during the period of the early impact of coronavirus disease (1). Nonetheless, this situation will become even more critical as the global measles crisis has killed thousands of unvaccinated children. Thus, the combination of measles and COVID-19 is very dangerous.

On the other hand, several countries did not achieve the immunisation coverage targets even before the pandemic COVID-19 began. Previous studies have found a sharp increase in vaccination refusal among parents, from 470 cases in 2013 to more than 12,000 cases in 2014 (3). Misinterpretations of disease severity, limited knowledge of vaccines, fears of adverse events and concerns over vaccine safety are often identified as major contributors to vaccine scepticism.

Misinterpretations of disease severity, limited knowledge of vaccines, fears of adverse events and concerns over vaccine safety are often identified as major contributors to vaccine scepticism (3, 7–15). Many parents also stated they were concerned because their children received too many vaccines in the first two years of life (9). They tend to rate higher the danger of side effects of the MMR vaccine and they are more concerned about the link associations between autism and MMR vaccine.

Moreover, parents deliberately refuse to vaccinate their children and prefer natural remedies or alternative medicines to prevent vaccine-preventable diseases (3, 8, 12, 16–18). As a result, if action is untaken to push the immunisation programme back right on target, we may barely see that children are affected by diseases where the disease was previously under control. For example, the Hand, Foot and Mouth Disease outbreak that happens in Sarawak took live 29 children because of enterovirus 71 widespread within the community (19). Several studies have been conducted towards knowledge and practice regarding children’s vaccination in Malaysia. However, a limited study specifically focuses on MMR vaccination. Therefore, this study was carried out to determine the parents’ knowledge and practice regarding MMR vaccination and examine its relationships.

## Methods

This study was conducted cross-sectional to collect information about the parents’ knowledge and practice regarding MMR vaccination. A cross-sectional design was selected because this current study was conducted at one point in time, and therefore, the variables are measured simultaneously in a given population (20). This study was conducted in a health clinic in the sub-urban residential in Selangor, Malaysia using simple random sampling. The total number of respondents in the health clinic is 202 (52 males and 150 females). A study was carried out from February 2020 to March 2020. In this study, simple random sampling was used due to it is simple to use, time-consuming and easy to assess the sampling error. In this study, the sample size was calculated using the formula of Krejcie and Morgan in 1970. With an accepted margin of error of 5% and a 95% confidence interval, the sample size required was 217 participants. Furthermore, eligible participants have included Malaysian parents with a toddler 1-year-old and literate in Bahasa Malaysia or English. Those parents with an immunosuppressive child were excluded from this study. For a respondent who fulfils the criteria, an explanation was given on the objectives and the respondent obtained written consent.

This study measured the parents’ knowledge and practice regarding MMR vaccination using the modified self-administered questionnaire developed by Awadh et al. (12). This part is also a close-ended question, consists of 10 questions that aim to assess and evaluate parents’ level of knowledge regarding MMR vaccination that was modified from the literature we chose. In addition, some of the questions are modified to fit our objective. The original questionnaire is more generalised to access childhood immunisations, but our focus is only on MMR immunisations. In this part, participants will be needed to choose only one answer provided for every 10 questions, whether ‘yes’ or ‘no’. One point will be given if the participants choose the correct answer and 0 point for the wrong answer. Therefore, the possible total knowledge score will be 0–10 points. The result will measure the level of knowledge into three categories: low, moderate and high. Cut off points to grade the categories are categorised into low (0–4 points), moderate (5–7 points), and high (8–10 points).

The last part of the instrument aims to assess and evaluate parents’ level of practice regarding MMR vaccination by providing 10 close-ended questions. In this part, participants will be needed to choose only one answer provided for every 10 questions, whether ‘yes’ or ‘no’. One point will be given if the participants choose the correct answer and 0 point for the wrong answer. Thus, the possible total practice score will be 0–10 points. The result will measure the level of practice into two categories which are poor and good. Cut off points to grade the categories are into good practice (6–10 points) and poor practice (0–5 points). The questionnaire was translated back-to-back to Bahasa Malaysia, and the translate tool demonstrates a good internal consistency of 0.82 with a similar Cronbach’s alpha. In this study, the questionnaire was distributed to bilinguals, Bahasa Malaysia and English.

### Ethics, Consent and Permission

Ethical approval was obtained from the research ethics committee prior to conducting the study. In addition, all participants provided written informed consent prior to participation.

### Statistical Analyses

The data were analysed using SPSS Statistic for Windows (version 23.0), in accordance with the study’s aim and the characteristics of the variables. The significance level was set at *P* < 0.05. The descriptive analyses were conducted to indicate the distributions of the demographic and inferential analyses like chi-squared test were used. All information was presented in tables.

## Results

### Demographic Characteristics

The result of this study revealed the 202 respondents were involved in this study. Out of that, 74.3% are females and 25.7% are males. The majority of respondents had 2–3 children (59.9%), Malay (70.8%), Islam (70.3%) and obtained a tertiary level of education (53%). Moreover, 102 (50.5%) of respondents had monthly family income between RM2,001–RM4,000 and most of them are working. The characteristics of the respondent’s sociodemographic data are presented in Table 1.

### Parent’s Knowledge and Practice of Measles-Mumps-Rubella Vaccination

In Table 2, the results of this study showed that 53.5% of the respondents had low knowledge of MMR vaccination, 35.1% had moderate knowledge and 11.4% had high knowledge of MMR vaccination. Meanwhile, the respondent with a poor scoring of MMR vaccination practice is 45.4% (92) and 54.5% (110) had good practice of MMR vaccination.

### The Association between Parents’ Knowledge and Practice Regarding Measles-Mumps-Rubella Vaccination

In this study, the association between parents’ knowledge and practice regarding MMR vaccination was examined, and the results of analyses were tabulated in Table 3 based on univariate analysis. The result of this study revealed a low level of knowledge of MMR vaccination among females (50.0%), working (52.1%), Muslim (53.8%), Malay (53.8%) who had an education until tertiary level (57.9%) and have only one child (41.7%). The result also reported that 18.3% of respondents who had one child were noted to own a high knowledge of MMR vaccination. Based on the results presented in Table 2, a moderate level of knowledge of MMR vaccination was reported among the respondents who had one child (40%), a primary education level (56.3%) and a working family (38.7%). Moreover, the respondents who had a tertiary education level (18.7%) and not a working family (20.5%) reported high knowledge of MMR vaccination.

Besides, there was a significant association between the level of knowledge on MMR vaccination with parental education background (*P* = 0.002), number of children (*P* = 0.040) and a working family (*P* = 0.030). Table 3 showed the number of respondents who had a higher level of knowledge and good MMR vaccination practices was 20.0%. Meanwhile, 84.8% of respondents who had poor practice also had a low level of knowledge on MMR vaccination. About 52.7% of respondents with a moderate level of knowledge reported having a good MMR vaccination practice. There is a significant association between MMR vaccination’s parental practice and the level of knowledge of MMR vaccination (*P* > 0.001).

The result showed that MMR vaccination’s poor practice is among the respondents’ who are male (57.7%) and had more than two children (50%). About 46.9% of Muslim, Malay and working respondents (43.6%) also had a poor practice of MMR vaccination. Meanwhile, the respondents who had a good practice of MMR vaccination are female (58.7%), had one child (65.0%), non-Muslim and non-Malay (57.6%), and working families (56.4%) were reported to have a good practice of MMR vaccination. The Pearson’s chi-squared test showed a significant association between gender and level of practice of MMR vaccination (*P* = 0.040). Besides, the number of children also found a significant association with their MMR vaccination practice (*P* = 0.050).

## Discussion

This cross-sectional study assessed the level of knowledge and practice towards MMR vaccination among parents in sub-urban residential in Bandar Seri Putra, Selangor, Malaysia. Overall, most parents have low knowledge of MMR vaccination but more than half of the respondents have good practice regarding MMR vaccination. This shows that even though most parents have low knowledge, it cannot indicate that parents would not be able to provide a positive contribution to their children’s health. Moreover, the association between levels of knowledge on MMR vaccination was significant with the practices of MMR vaccination. However, if we analysed more detail, the differences between all levels are minimal in numbers. The number of parents who had high knowledge of MMR vaccination was good in practice too. Hence, this situation demonstrates the parents’ ability to resolve all doses of MMR vaccine for their children (12, 21–22). This finding is comparable to previous studies (23). In contrast, even though parents used immunisation as a technique to guard their children against vaccine-protected disease, their knowledge, likewise their understanding of MMR vaccination is still limited, which may lead to uncompleted immunisation status of children (9–10, 14).

The result of this study revealed that most parents have good practice on MMR immunisation and majorities of them are females, Muslim, Malay and working families. This shows that they did alert with their children immunisation schedule that was resulting in their practice regarding immunisation. The finding was consistent with a previous study where most mothers whose children received partial or no measles immunisation did not know about the measles immunisation schedule. Since there is a small percentage difference between good and poor practice, it may be due to certain parents’ self-attitude towards their children immunisation schedule.

Furthermore, in this study, there is a significant association between the level of knowledge on MMR vaccination and the demographic characteristics such as parent’s education level and the number of children. The result of this finding was similar to the previous study in which parents with higher education levels had better knowledge regarding MMR vaccination (3, 8, 17). In contrast, the result of this study revealed the parent’s education level did not influence the practice of MMR vaccination. Nevertheless, a study conducted by Borr’s et al. (24) found that parents with a high education level had higher vaccination coverage.

Previous research found that children of university-educated parents had less probability of being vaccinated and sometimes they delayed the administration of vaccines compared to parents with lower educational levels (11). This result was contrasted with Mabrouka’s (14) study, where they revealed no significant relationship between immunisation status and parents’ educational level. These inconsistent findings may be due to the parents’ different types of information sources regarding MMR vaccines and the importance of immunisation schedules, especially MMR vaccination. Therefore, the social media mass, the internet and the healthcare professionals are crucial in transferring good knowledge about MMR vaccination to the parents (8–10, 17). In the era of technological advances, there is a great resource for parents to easily access information regarding MMR vaccination through mass media, either by surfing the internet through computers or smartphones. Mass media’s success in the field of commerce is often considered appropriate to influence parent’s behaviour towards health and modifying pre-existing patterns of behaviour or attitudes to enhance optimum health. However, sometimes parents are easily influenced by false information as well.

In this study, most parents who had high knowledge are among the parent who had one child compared to parents who had more than one child. Indeed, there was a significant relationship between the number of children and parents’ knowledge of MMR vaccination. However, the parents who had more children have good practice towards MMR immunisation compared to parents who had one child. This scenario may be influenced by the experienced they had before, as well as they had received information beforehand, especially about side effects and influence from anti-vaccine (11). In contrast, a previous study reported no relationship between the number of children and knowledge of MMR vaccination (25).

This study had its strength, which helps to know the factor influencing parent knowledge-practice on MMR vaccination. Thus, the findings may help make evidence-based in strengthening health promotion, especially on increasing the MMR vaccination uptake. However, this study also had its limitations. First, it uses a cross-sectional study design that seems to produce information about this situation but cannot explore the direction of an association or causal of vaccination practice. Thus, we cannot predict whether the parents with good practice in MMR vaccination comply with the vaccination schedule for their children within the future. Therefore, the results of this study cannot be generalised to other parents in Malaysia. Secondly, due to the nature of the self-administered questionnaire, data on vaccination status were not validated by other data sources, and reporting biases may be introduced. Besides that, this study was considered local data collected among sub-urban residents in Selangor, Malaysia. Therefore the results cannot be generalised for the whole Malaysian community.

Qualitative studies have been proposed for future research to explore vaccine hesitation issues that have not yet reached in-depth information on parental perceptions of MMR vaccination. Moreover, an intervention study was also recommended evaluating the current health promotion of childhood immunisation, especially MMR vaccination.

## Conclusion

This study showed that most parents have low knowledge of MMR vaccination; however, they have a good practice of MMR vaccination. Furthermore, the educational level and the number of children were significantly related to parents’ knowledge of MMR vaccination. Thus, the parent’s educational level needs to be taken into consideration when planning the health promotion on vaccinations and awareness programmes to enhance the knowledge about the advantages and importance of vaccination, especially in Selangor and other sub-urban areas.

## Figures and Tables

**Table 1:** The sociodemographic characteristics, level of knowledge and practice on MMR vaccination (*n* = 202)

Variable	n (%)	Mean (SD)
Gender		1.74
Male	52 (25.7)	(0.44)
Female	150 (74.3)	
Age		31.0
20–29	79 (39.1)	(4.48)
30–39	118 (58.4)	
> 40	5 (2.5)	
Number of children(s)		1.81
1	60 (29.7)	(0.61)
2–3	121 (59.9)	
> 3	21 (10.4)	
Race		1.59
Malay	143 (29.0)	(1. 00)
Indian	70.8 (14.4)	
Chinese	14 (6.9)	
Others	16 (7.9)	
Religion		1.72
Islam	143 (70.3)	(1.17)
Hinduism	22 (10.9)	
Buddhism	6 (3.0)	
Others	32 (15.8)	
Education level		3.45
Primary school	16 (7.9)	(0.64)
Secondary school	79 (39.1)	
Tertiary education	107 (53.0)	
Employment status		2.48
Self-employed	53 (26.2)	(1.15)
Private	55 (27.2)	
Government	39 (19.3)	
Unemployed	55 (27.2)	
Family income		2.46
< RM2,000	4 (2.0)	(0.54)
RM2,001–RM4,000	102 (50.5)	
> RM4,000	96 (47.5)	

**Table 2:** The parent’s knowledge and practice of MMR vaccination (*n* = 202)

Variables	Frequency (%)	Mean (SD)
Level of knowledge of MMR vaccination		0.58 (0.67)
High knowledge	23 (11.4)	
Moderate knowledge	71 (35.1)	
Low knowledge	108 (53.5)	
Practice of MMR vaccination		0.54 (0.50)
Good practice	110 (54.5)	
Poor practice	92 (45.4)	

**Table 3:** The association between sociodemographic characteristics, level of knowledge and level of practice towards MMR vaccination (*n* = 202)

Variables	Level of knowledge of MMR vaccination			*χ* ^2^	*P*-values	Level of practice of MMR vaccination		*χ* ^2^	*P*-values
	
	Low *n* (%)	Moderate *n* (%)	High *n* (%)			Good *n* (%)	Poor *n* (%)		
Gender								4.17	0.040*
Male	33 (63.5)	15 (28.8)	4 (7.7)	2.94	0.230	22 (42.3)	30 (57.7)		
Female	75 (50)	56 (37.3)	19 (12.7)			88 (58.7)	62 (41.3)		
Education level								0.60	0.440
Secondary level and below	55 (57.9)	37 (38.9)	3 (3.2)	**12.06**	**0.002** *	49 (51.6)	46 (48.4)		
Tertiary education	53 (49.5)	34 (31.8)	20 (18.7)			61 (57.0)	46 (43.0)		
Number of children								3.83	0.050*
1	25 (41.7)	24 (40.0)	11 (18.3)	**6.41**	**0.040** *	39 (65.0)	21 (35.0)		
> 2	83 (58.4)	47 (33.1)	12 (8.5)			71 (50.0)	71 (50.0)		
Religion								0.34	0.560
Muslim	77 (53.8)	46 (32.2)	20 (14.0)	4.16	0.120	76 (53.1)	67 (46.9)		
Non-Muslim	31 (51.6)	25 (41.7)	4 (6.7)			34 (57.6)	25 (42.4)		
Race								0.34	0.570
Malay	77 (53.8)	46 (32.2)	20 (14.0)	4.16	0.130	76 (53.1)	67 (46.9)		
Non-Malay	31 (51.7)	26 (43.3)	3 (5.0)			34 (57.6)	26 (42.4)		
Working								1.34	0.250
Working	85 (52.1)	63 (38.7)	15 (9.2)	**6.75**	**0.030** *	92 (56.4)	71 (43.6)		
Not working	23 (59.0)	8 (20.5)	8 (20.5)			18 (46.2)	21 (53.8)		
Practice of MMR vaccination									
Poor practice	78 (84.8)	13 (14.1)	1 (1.1)	**67.96**	**> 0.001** *				
Good practice	30 (27.3)	58 (52.7)	22 (20.0)						

Note:

* *P* < 0.05
